# *Triatoma williami* in intradomiciliary environments of urban areas in Mato Grosso State, Brazil: domiciliation process of a wild species?

**DOI:** 10.1186/s40249-022-00938-4

**Published:** 2022-02-14

**Authors:** Mirian Francisca Martins, Sinara Cristina de Moraes, Jader Oliveira, Janaina Cipriana dos Santos, Ludier Kesser Santos-Silva, Cleber Galvão

**Affiliations:** 1 Department of Environmental Health Surveillance, State Health Secretary of Mato Grosso-SESMT, Amaro Leite 474, Barra do Garças, MT 78600-027 Brazil; 2 Public Health Faculty, Public Health Entomology Lab, University of São Paulo, São Paulo, SP Brazil; 3 Institute of Biological and Health Science, Federal University of Mato Grosso, Barra do Garças, MT 78600-000 Brazil; 4 National and International Triatomini Taxonomy Reference Lab, Instituto Oswaldo Cruz-IOC, FIOCRUZ, Av. Brasil 4365, Pavilhão Rocha Lima, Sala 505, Rio de Janeiro, RJ 21040-360 Brazil

**Keywords:** Triatominae, *Triatoma williami*, Colonization, *Trypanosoma cruzi*, Chagas disease, Surveillance program

## Abstract

**Background:**

Triatomines in Latin America are natural Chagas disease (ChD) vectors. Triatomine domiciliation is one of the main factors increasing the occurrence risk of this disease in humans. There are 66 triatomine species in Brazil, with three genera of significant epidemiological importance—*Panstrongylus*, *Rhodnius,* and *Triatoma*. Among the *Triatoma* species, *Triatoma williami,* a wild species, has been reported in Goiás, Mato Grosso, and Mato Grosso do Sul. In the Barra do Garças, Mato Grosso, the invasion by triatomines has been reported, with *T. williami* being the most common species. This study aimed to survey triatomine fauna and determine the *Trypanosoma cruzi* natural infection rates in triatomines in the urban area of Barra do Garças, Mato Grosso, Brazil.

**Methods:**

Triatomine specimens were sampled by passive surveillance or active search by agents combating endemic diseases from 2019 to 2020. A parasitological feces diagnosis was performed to detect the presence of *T. cruzi* after the specimens were identified. Concerning *T. cruzi* identification, molecular diagnosis and genetic sequencing were performed to determine the strain, also called discrete typing units (DTUs).

**Results:**

The 211 triatomines were collected, distributed in specimens of *T. williami* (84.4%), *P. geniculatus* (3.3%), *P. diasi* (1.4%), and *R. neglectus* (10.9%). Two colonies of *T. williami* were found through morphological analyses. These insects were sampled inside domiciles in an urban area neighboring Jardim Pitaluga (15° 51′57.7″ N, 052° 16′ 04.5 E). The records were sampled in September 2019 and January 2021. The rate of natural infection by *T. cruzi* was 39.4%. Two *T. williami* specimens from the sampled colonies were positive for the *T. cruzi* strain DTU IV.

**Conclusions:**

This is the first time that *T. williami* has been confirmed in an urban area of Barra do Garças, Mato Grosso, Brazil. Further studies are needed for a clearer understanding of the ecology of this species for prevention and control mechanisms since its sampled specimens had a high rate of natural infection by *T. cruzi*.

**Graphical Abstract:**

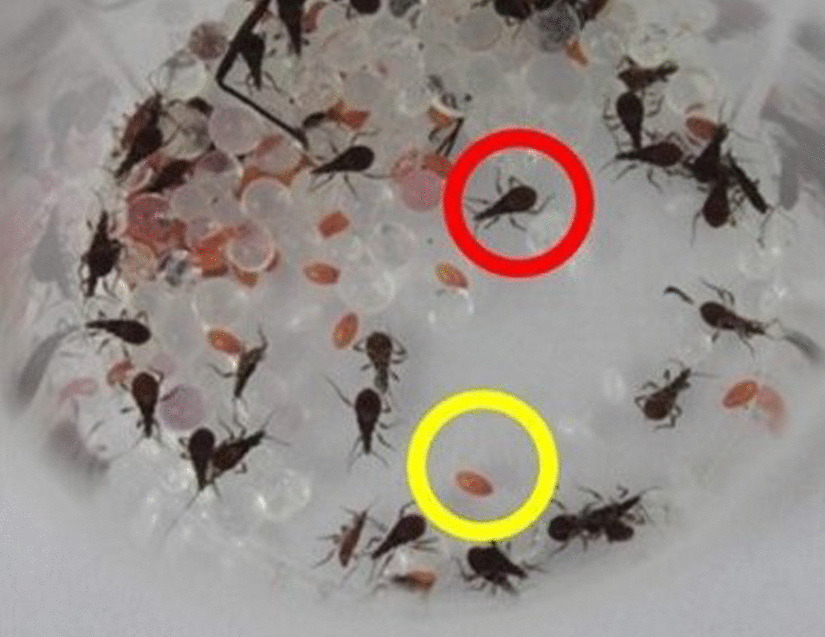

**Supplementary Information:**

The online version contains supplementary material available at 10.1186/s40249-022-00938-4.

## Background

The blood-sucking Triatominae subfamily (Hemiptera, Reduviidae) are vectors of *Trypanosoma cruzi* (Chagas, 1909) (Kinetoplastida, Trypanosomatidae), which causes Chagas disease (ChD). A total of 154 extant and three fossil species are comprised within five tribes and 18 genera of this subfamily [1]. In Brazil, 66 triatomine species have been previously recorded [2–6]. Three genera have particularly significant sanitary importance and are related to the transmission of *T. cruzi* to humans: *Panstrongylus*, *Rhodnius*, and *Triatoma* [2, 7–10].

Vectorial transmission by *Triatoma infestans* was officially eliminated from Brazil in 2006, and efforts were made to halt the transmission of ChD by non-native vectors [11]. Despite this significant achievement, with such a “ChD vector elimination certification”, a false idea may be given that disease transmission does not exist [12]. Native triatomine species are potential vectors of *T. cruzi* and can invade and colonize artificially in domestic and peridomestic environments [13].

Triatominae domestication may be envisaged as an extension of the evolutionary route from predator to nest-dwelling bloodsucker, where the domestic habitat represents a particular type of vertebrate ‘nest’ [14]. The infestation and density index have often been considered indicators of the level of intrusion of a species into the domestic habitat, while the colonization index can be viewed as a measure of its domiciliation or domestication [15]. Domiciliary species are characterized by developing their life cycle inside domiciles or peridomicile structures. This assumption will be confirmed if adults, nymphs, eggs, and exuviae are caught [15].

Triatominae domiciliation is one of the main factors in the increasing transmission risk of *T. cruzi* to humans [16]. Anthropogenic morphoclimatic alterations propel the domiciliation process and dispersal of triatomines and can be interpreted as a survival strategy for the species [17]. Silveira et al. [18] reported that the domiciliation process results from factors that may promote the invasion and progressive adaptation of triatomines to the human domicile and may be related to the environment and the inherent characteristics of the vector. The domiciliation could be accelerated by the opportunism of wild triatomine species in the scenario of natural food source scarcity. The human demographic shift is partially responsible for the changes in vectorial transmission. With such a shift promoting unfavorable environmental changes and subsequent rarefaction in wild fauna, triatomines are led to move to highly artificial habitats where various hiding places, stability, and abundance of food throughout the year exist [19].

The trends toward domesticity seem to be associated with behavioral plasticity, reducing the genetic repertoire of specimens, and increasing developmental instability. These conditions make triatomine species and populations more efficient vectors [19, 20].

For a long time, triatomine species have been classified according to their adaptation to human domiciles. This classification usually occurs in three groups, with triatomine species classified as domiciliary, peridomiciliary, and wild. Noireau et al. [21] proposed four categories of adaptation of triatomine species to human domiciles: sylvatic, intrusive, domiciliary, and domestic species. Such definitions have been the most widely accepted and are used in the literature to classify many triatomine species [15].

From the vector control perspective, it is of significant importance to determine precisely three aspects of the relationship between triatomines and humans: (i) the prevalence of sylvatic populations of triatomines, (ii) the level of intrusion of these sylvatic populations in peridomicile and inside domicile, and (iii) the domiciliation or domestication levels in peridomicile and domicile [15]. Nowadays, attention has been focused on the domiciliation of species, considered sylvatic because of increasing reports of wild species invading the domicile and peridomicile in South American countries [22–24]. Invasions by at least 10 species have been reported in the literature in the last few decades, increasing the risk of intradomiciliary colonies in South American countries, such as Brazil, Bolivia, Colombia, and Venezuela (Additional file 1: Table S1).

*Triatoma williami* Galvão, Souza e Lima, 1965 is a wild species reported in the Brazilian states of Goiás, Mato Grosso, and Mato Grosso do Sul [2, 25, 26]. The risk of domiciliation of this species was previously hypothesized by Andrade-Neto et al. [27] through interviews with residents. Here, the domiciliation of this species was demonstrated by observing all of its life stages, including eggs, within the domiciles.

This study aimed to survey triatomine fauna and determine the natural infection rates in Barra do Garças municipality, Mato Grosso State in Brazil. This study would provide relevant information for improving the ChD monitoring activities of sanitary authorities.

## Methods

### Study area

**Fig. 1:**
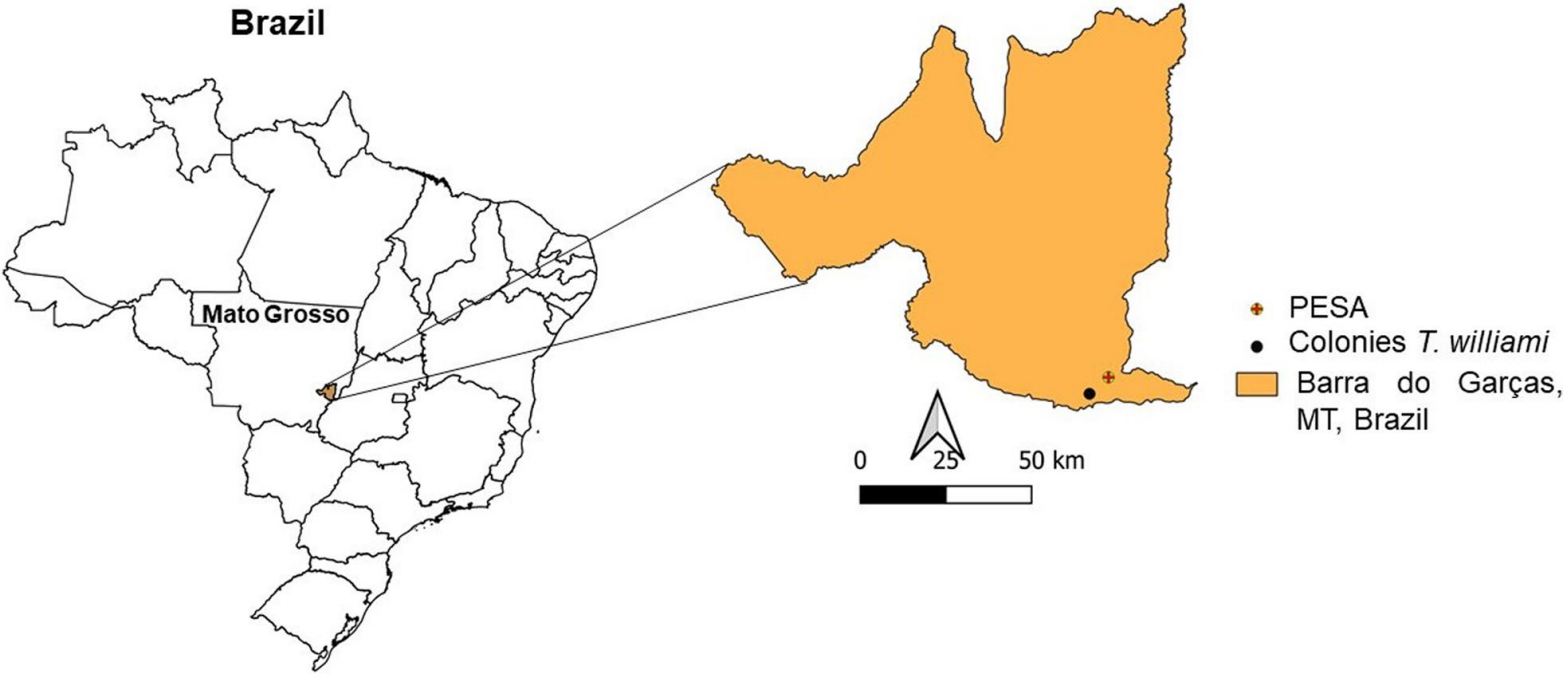
Municipality of Barra do Garças, Mato Grosso, Brazil. Adapted from the Brazilian Institute of Geography and Statistics (Instituto Brasileiro de Geografia e Estatística—IBGE), geographic coordinates (15° 51′57.7″ S, 052° 16′04.5″ W) from the *Triatoma williami* colonies and the Parque Estadual da Serra Azul (PESA) (15° 49′15″ S 52° 12′57″ W)

This study was conducted in an urban area of Barra do Garças, Mato Grosso, Brazil. This municipality (15° 51.98ʹ S, 052°16.29ʹ W, 395 m altitude) is located in the eastern Mato Grosso region bordering the state of Goiás, also known as Vale do Araguaia. The municipality has a population of 56,423 inhabitants and a territorial area of 9079 km^2^ [28] (Fig. 1).

Barra do Garças has a different ecological context, given its proximity to the Parque Estadual da Serra Azul conservation unit facility (PESA abbreviation in Portuguese) [29]. PESA (15°49′15″ S 52°12′57″ W) has 11.002 ha, with several Brazilian Cerrado phytophysiognomies (e.g., gallery forests, semi-deciduous forests, typical cerrado, and predominantly rupestrian Cerrado) [30] (Additional file 2: Fig. S1).

The Brazilian Cerrado biome is characterized by spatial heterogeneity and high plant and insect endemism [31, 32]. This biome is considered a world biodiversity hotspot. The climate is characterized by two seasons, dry winters and rainy summers, corresponding to the Aw climatic type, according to the Köppen classification [33]. Annually, the climate in this region has a 60% annual average humidity and an annual average temperature of 20–27 °C.

### The health organization

The headquarters of the municipality are organized in 53 neighborhoods. The city health secretary has 45 endemic control field agents to perform health surveillance and vector control activities. Three of these agents work in the surveillance and control of ChD vectors. The action of these agents consists of performing health education for ChD prevention and domicile inspections, both in the inner and the peridomicile. If triatomine specimens are found, chemical control is performed using residual action insecticides made available by the Brazilian Ministry of Health.

Barra do Garças is comprised in the health region Garças Araguaia. This city is the location of the regional health office of Barra do Garças and the Entomology Laboratory at the Regional Health Office (ERSBG abbreviation in Portuguese) from the state health secretary of Mato Grosso (SESMT abbreviation in Portuguese). Monthly spreadsheets from the ChD control program (PCDCh abbreviation in Portuguese) containing data on the activities were sent to ERSBG by SESMT. With this information, PCDCh was monitored by SESMT. The most frequent specimens, vectorial density, and natural rate of infection were analyzed in ERSBG. Investigations of whether there were seasonal patterns for species and the construction of historical series, among other activities, were also conducted by ERSBG.

### Taxonomic identification of insects

The analyzed insects were collected via passive surveillance by residents or active search by agents combating endemic diseases on scheduled domicile visits from 2019 to 2020. The second colony was found in January 2021 and entered in this analysis without waiting for the ERSBG database to close at the end of each year.

The inhabitants usually collected triatomine specimens found in their domiciles. These people informed the health workers or delivered the specimens directly to the ERSBG. All the sampled material was analyzed in this facility. Specimen identification was made using dichotomous keys [2, 16]. The identification of these insects as *T. williami* was conducted based on the external morphological characteristics of the head, wing, abdomen, and external female genitalia (Additional file 3: Fig. S2A–E).

### Natural infection by *T. cruzi*

#### Parasitological diagnosis

A parasitological examination of fresh feces from live-sampled specimens with intestinal contents was performed. For this purpose, 5 μl of intestinal content plus two drops of phosphate-buffered saline (pH 7.2) between slides and coverslips were observed under a microscope optical at 400×. This procedure is the conventional diagnosis recommended by the Ministry of Health in Brazil.

#### Molecular diagnosis

For the analysis of natural infection by *T. cruzi*, conventional polymerase chain reactions (PCR) were performed, as previously described by Brígido et al. [34] using oligonucleotides P21 fw (5′-AACGCACCATCAATCTTTTG-3′) and P21 rv (5′-CGTCGCATTCCTCATTTCTTC-3′) that amplify a 65 bp fragment from the genomic region of the parasite. For the discrete type of unit (DTU) identification of *T. cruzi*, PCR was performed according to Consetino et al. [35]. For this analysis, oligonucleotides TcSC5D-forward 5′-GGACGTGGCGTTTGATTTAT-3′ and TcSC5D-reverse 5′-TCCCATCTTCTTCGTTGACT-3′ were used. An amplicon of 832 bp from the TcSC5D gene was produced with these oligonucleotides. The amplicons were sequenced using an ABI3730xl Genetic Analyzer (Applied Biosystems: Life Technologies Corporation, 5791 Van Allen Way, Carlsbad, California 92008). The *T. cruzi* TcSC5D gene sequences were analyzed using the Clustal Omega tool [36] to discriminate the DTUs TcI from TcVI using the polymorphic sites described by Consentino et al. [35].

### *T. williami* domiciliary colonization

In September 2019, a request from a resident was attended by the ChD surveillance team, where a triatomine entomological survey was conducted. In one bed within this domicile, the field agents found the first *T. williami* colony containing adults, first instar nymphs, and eggs.

In January 2021, the agents performed another entomological research in that domicile. This time, the agents found adults, nymphs of different stadium, and eggs. As recommended, the agents carried out residual chemical control in this domicile, and subsequent more *T. williami* specimens from different stages of development were found.

### Data analysis

The data were tabulated and analyzed using Excel 2016 for Windows® (Microsoft, Redmond, USA). The following entomological indicators were considered in the analysis: number of triatomines captured (males, females, and nymphs), number of triatomines found inside and outside of the domiciles, and number of infected triatomines.

Descriptive statistics were used to obtain the absolute and relative frequencies. The absolute frequency was estimated as the number of triatomine collected by specie and to calculate the relative frequencies used the absolute frequencies by the total number of triatomines.

Only entomologic indices are considered risk factors associated with *T. cruzi* transmission. The results are presented in percentages and indices.

The triatomine prevalence was estimated as the number of triatomine specimens by the total number of triatomines collected. The natural infection rate was calculated from the number of infected triatomines/number of triatomines examined × 100.

## Results

### Triatomine species invading dwellings in the urban area of Barra do Garças

A total of 211 triatomines were collected during this study, 155 in 2019 and 56 in 2020. Specimens of *T. williami* (84.4%), *P. geniculatus* (3.3%)*, P. diasi* (1.4%), and *R. neglectus* (10.9%) were found during the surveys. Since 2013, there have been records of triatomines invading domiciles in urban areas in Barra do Garças, Mato Grosso, Brazil (Additional file 4: Fig. S3).

*T. williami* has been registered in eight neighborhoods in the urban area of Barra do Garças, Mato Grosso, Brazil. In 2019 and 2020 (Table 1), the highest infestations recorded were found in boundaries neighborhoods of the PESA in descending order: Jd. Amazônia I–BNH, Jd. Pitaluga, Jd. Araguaia and Santo Antônio.

**Table 1:** Distribution of the collected *Triatoma williami* specimens by neighborhood in Barra do Garças, Mato Grosso, Brazil, in 2019 and 2020

*T. williami* abundance by neighborhood	2019		2020	
	AF	RF (%)	AF	RF (%)
**Jd. Amazônia I—BNH**	57	41.6	32	**78.0**
**Jd. Pitaluga**	72	52.6	9	**22.0**
**Jd. Araguaia**	2	1.5	0	0.0
**Santo Antônio**	2	1.5	0	0.0
Monte Sinai	1	0.7	0	0.0
Jd. Amazônia II	1	0.7	0	0.0
Jd. Piracema	1	0.7	0	0.0
São Sebastião	1	0.7	0	0.0
Total	137	100.0	41	100.0

Places in bold: boundary neighborhoods of the PESA

*AF* absolute frequency, *RF* relative frequency

### Population structure and environments occupied by *Triatoma williami*

The adult *T. williami* population was 75 females and 19 males in 2019 and 30 females and 10 males in 2020. The female-to-male ratio was not within the expected 1:1 pattern: 3.9 (75/19) and 3 (30/10) times more females than males. For immatures, we had 41 first-stage nymphs, one second-stage nymph, and one-fifth-stage nymph in 2019. In 2020, we had one first-stage nymph, one second-stage nymph, two fourth-stage nymphs, and one fifth-stage nymph.

From 2019 to 2020, 178 *T. williami* were collected. Sixty percent of them were found inside the domiciles, 22% in the peridomicile, and we did not have any information for 18% of them. For intradomicile environments, bedrooms and living rooms were the most infested (Table 2).

**Table 2:** The percentage of *Triatoma williami* collected from 2019 to 2020 in Barra do Garças, Mato Grosso, Brazil, from 2019 to 2020 in each environment

	Number of *Triatoma williami*			
Environment	2019	2020	Total	%
Indoor				
Bedroom	60	6	66	37.1
Living room	15	8	23	12.9
Kitchen	11	3	14	7.9
Bathroom	2	0	2	1.1
	88	17	105	59
Peridomicile				
Balcony	29	6	35	19.7
Garage	1	0	1	0.6
Barbecue grill	1	0	1	0.6
Upper balcony	0	3	3	1.7
Subtotal	31	9	40	22.5
No information	18	15	33	18.5
Total	137	41	178	100

**Fig. 2:**
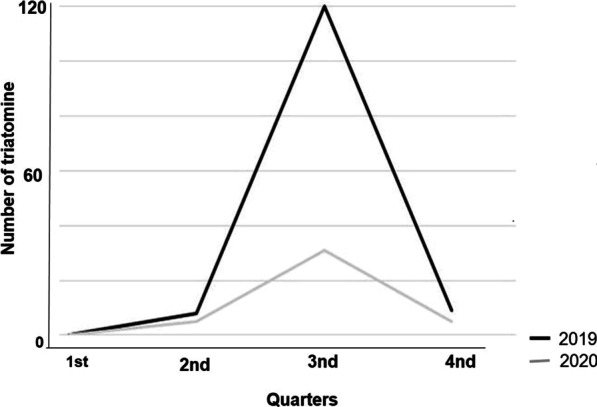
The number of *Triatoma williami* collected between 2019 and 2020 in Barra do Garças, Mato Grosso, Brazil

We observed a seasonality pattern from a monthly density analysis of the sampled triatomines from 2019 to 2020. Such a pattern was associated with the increase in the sampled triatomine in the third quarter of the respective years analyzed. This period was recorded as the driest climatic season in the region (Fig. 2).

### Natural infection by *T. cruzi*

A total of 211 triatomine specimens were collected between 2019 and 2020. It was possible to assess the natural infection in 132 specimens from these samples. Of the 132 triatomine specimens analyzed, 52 (39.4%) were naturally infected with *T. cruzi,* revealing a high natural infection rate. *T. williami* had a higher infection prevalence (Table 3). Four triatomine species were found naturally infected. The natural infection rate in descending order according to species was 43.6% (48/110), 33.3% (1/3) for *P. diasi* and *P. geniculatus,* and 12.5% (2/16) for *R. neglectus*. *T. williami* had the highest infection prevalence, which reached 92% (48/52), representing the most significant risk for the human population in Barra do Garças.

**Table 3:** The natural infection for *Trypanosoma cruzi* according to triatomine species collected in Barra do Garças, Mato Grosso, Brazil, for 2019 to 2020

Species	No. of specimens analyzed	No. of positive	Infection rate (%)	Infection prevalence (%)
*Triatoma williami*	110	48	43.6	92.3
*Rhodnius neglectus*	16	2	12.5	3.8
*Panstrongylus diasi*	3	1	33.3	1.9
*P. geniculatus*	3	1	33.3	1.9
**Total**	132	52	39.4	100.0

Data recorded in the monthly sheet of the ChD Control Program, SESMT.Brazil. Unpublished data

### *T. williami* in the process of domiciliary colonization

**Fig. 3:**
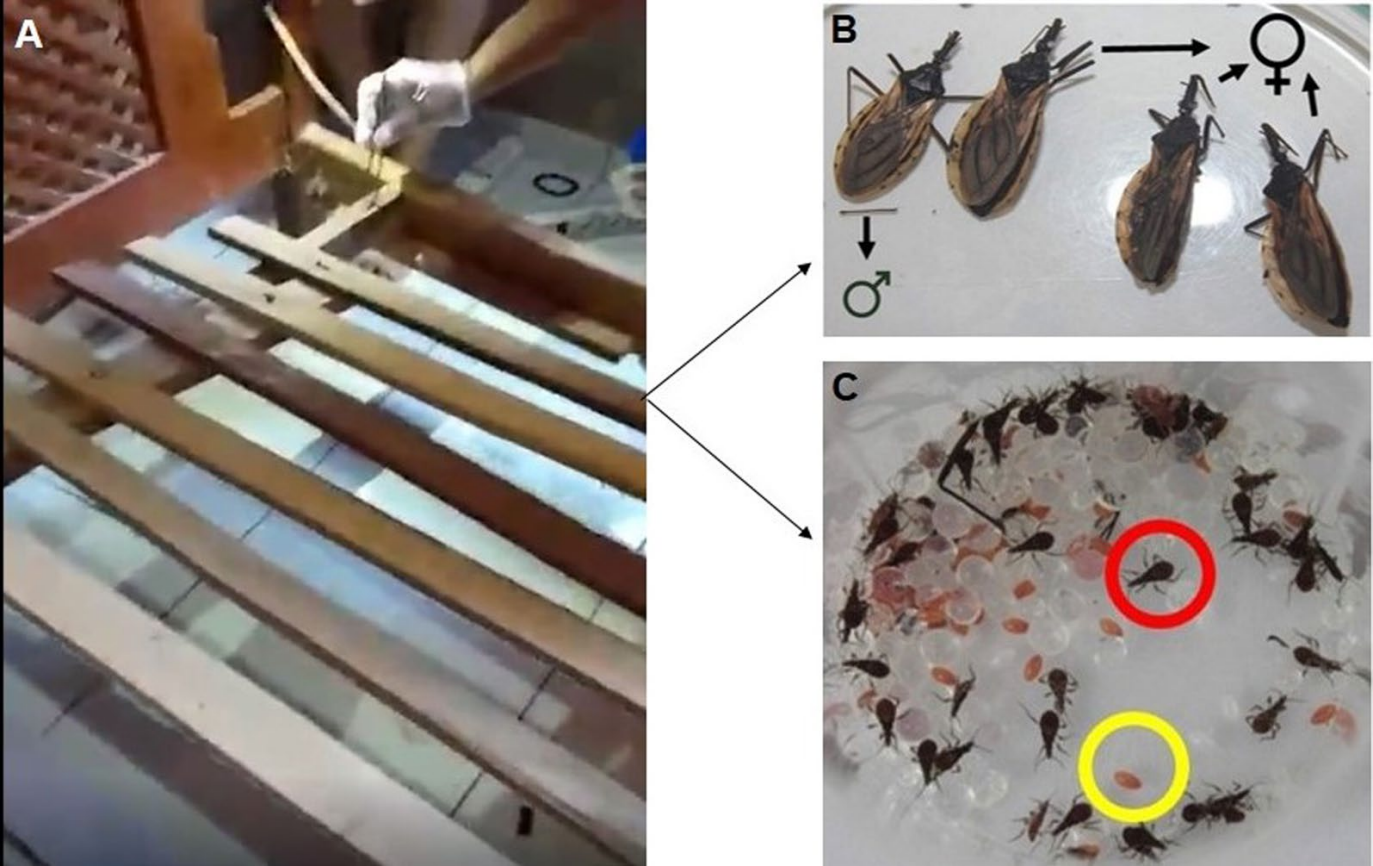
**A** The field agents performed an entomological survey in the cracks of resident bed; **B** One male and three female specimens of *Triatoma williami;* **C** Eggs (yellow circle) and nymphs (red circle) collected in Barra do Garças, Mato Grosso, Brazil, in 2019

In September 2019, in one searched bed, the field agents found four adult specimens (Fig. 3A, one male and three female), 34 eggs, and 41 nymphs in the first developmental stage (N1) on the wooden bed frame (Fig. 3B, C). Signs of feces were also observed.

After these triatomine specimens were found, the endemic control agents of the city health secretary from Barra do Garças conducted a residual chemical control in that domicile. In 2020, no entomological research or chemical control were performed in this domicile. In January 2021, more specimens were caught by residents inside their domiciles. These specimens were handed over to the ERSBG from the SESMT. This was the second compound domiciliation record with eight sampled specimens: two males, one female, and five nymphs (one nymph in the N1, one in the N2, two in the N4, and one in the N5 stages).

**Fig. 4:**
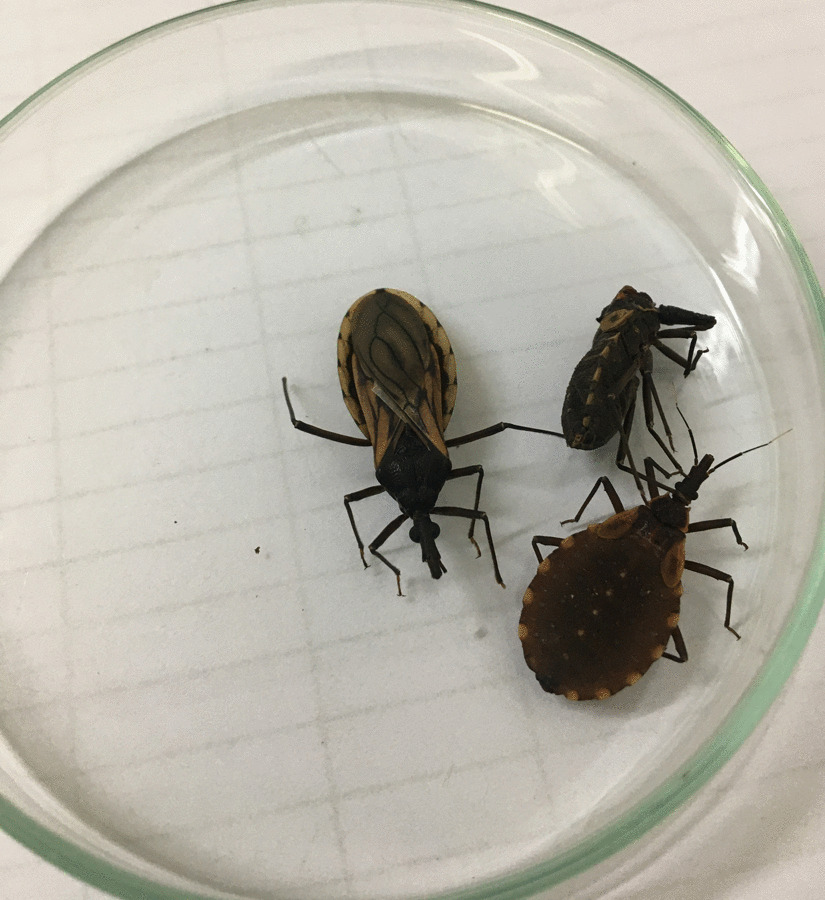
The specimens of *Triatoma williami* sampled after the residual chemical control in Barra do Garças, Mato Grosso, Brazil, in 2021

In February 2021, the field agents carried out residual chemical control in the infested domicile. In June 2021, the resident on that infested domicile collected and delivered two additional *T. williami* nymphs to the ERSBG from the SESMT: one nymph in the N1 stage, one dead male, and one exuviae (Fig. 4).

*T. williami* colonies were found in beds indoors in an urban area in the Jardim Pitaluga neighborhood (Fig. 1) in Barra do Garças, Mato Grosso, Brazil. Voucher specimens from the first colony were deposited in the Herman Lent Collection of the Instituto Oswaldo Cruz in Rio de Janeiro, Brazil, being two adults: CTIOC13005, CTIOC13006; three nymphs (CTIOC13007 to CTIOC13009) and five eggs.

Two adult specimens, one female and one male, and two nymphs from the second *T. williami* colony were deposited in the “Entomological Collection Prof. Dr. José Maria Soares Barata” in the Faculty of Pharmaceutical Sciences UNESP—Araraquara—São Paulo Brazil. Therefore, domiciliary colonization by two *T. williami* colonies found in an urban area in Barra do Garças, Mato Grosso, Brazil, was reported in this study.

### Identification of the* T. cruzi* strain

For the first colony, two adult female specimens were positive for *T. cruzi* in the parasitological diagnosis being submitted to the molecular diagnosis and later to the genetic sequencing. The DTU type IV of the *T. cruzi* strain was revealed by the identification results.

## Discussion

In a seven-year historical series, the invasion of wild triatomine species has been reported in urban domiciles from Barra do Garças, Mato Grosso, Brazil. *T. williami* was the primary specimen detected. The identification of *T. williami* was conducted based on external morphological characteristics. In such analyses, the importance of these structures in the identification of specimens was confirmed, as previously discussed by Teves et al. [37].

We reported the findings of all development stages of *T. williami*: adults, nymphs, and eggs inside human domicile from Barra do Garças, Mato Grosso, Brazil, during two different years. In the second year, we found nymphs in the last developmental stage, suggesting early stages in the domestication of this sylvatic species. The domiciliary colonization of wild triatomine species is a risk factor for reestablishing the vectorial transmission of ChD [15, 38]. The record of *T. williami* in the domiciliation process should be problematic because of the record of this species naturally infected by *T. cruzi* with a high infection rate.

So far, *T. williami* has been considered a secondary *T. cruzi* vector because it maintains its wild condition and shows synanthropic potential, colonizing the peridomicile and frequently invading the domiciles. Synanthropy represents a secondary adaptation by sylvatic species in response to environmental changes. Such adaptability to domiciles depends on triatomine plasticity [17]. One study [39] mentioned that morphological plasticity in the shape of *T. williami* is associated with blood source, but they did not test whether plasticity confers a fitness advantage to culminate in domiciliation by this species. However, a high potential vector for nymphs of this species was recorded in a previous study [40].

The first *T. williami* specimen in Nova Xavantina, Mato Grosso, Brazil, was recorded by Travassos-Filho (1972) [41]. This specimen was collected inside the domicile of a worker and infected with *T. cruzi*. Then Arrais-Silva et al. (2011) and Andrade-Neto et al. (2012) [26, 27], using molecular biology, found that the natural infection rate of this species by *T. cruzi* was 30% in Barra do Garças, Mato Grosso, Brazil. We observed an elevated infection rate of *T. williami* by *T. cruzi* in 2019 and 2020 in our parasitological feces diagnosis, indicating the transmission risk of the parasite by this vector.

The finding of the TcIV DTU of *T. cruzi* in *T. williami* collected at home is an unprecedented record for Mato Grosso, Brazil, and is worrisome. Although this DTU originates from the wild cycle, studies have already demonstrated its participation in domestic cycles [42, 43] that are frequently correlated with acute illnesses caused by ChD in humans. These cases are mainly associated with outbreaks of oral transmission of the parasite, with TcIV being one of the leading causes of human ChD in the western Brazilian Amazon [44–46]. Thus, the transmission risk of the parasite to humans in the region might be increased by the occurrence of triatomine colonies infected by this strain in domicile environments.

The distribution of DTU TcIV is the most important in Central and South America, with greater density in the Amazon region [42, 43]. In studies analyzing triatomines from different biomes, this DTU has not been recorded in the Cerrado [47], the predominant biome in our study area. With the finding of this strain in *T. williami*, we corroborate previous records related to the historical predominance of this DTU for the genus *Triatoma* [47].

Surveillance for species, considering the domiciliation principle, and the development of actions to interrupt the vector domiciliation process, is paramount in preventing ChD. The occurrence of wild triatomine species sporadically invading human domiciles is a significant difficulty in vector surveillance programs [48]. It is necessary to carry out immediate intervention to interrupt this domiciliation process of *T. williami* in the urban area of Barra do Garças, Mato Grosso, Brazil. The approximation of vectors with human living spaces increases the risk of transmission of ChD [49].

Analysis of the triatomines collected in Barra do Garças in a historical seven-year series showed earlier demonstrates the increase in vector density in 2019. Periods with a peak incidence of triatomines in the third quarter of the year are indicated by the results. Seasonality is also observed in Chagas vector populations and transmission. In combination with density-dependent regulation, these characteristics have led to the belief that insecticide control of these vectors can be improved if seasonally timed [50, 51].

When we analyzed the records of the locations with triatomines in Barra do Garças over seven years, we identified a higher occurrence in domicile in the four neighborhoods bordering PESA. Thus, the presence of a green belt associated with the artificial light of these domicile holds might support and promote the dispersion of triatomines. Martins et al. [29] reported that the proximity of Barra do Garças to PESA may facilitate human contact with triatomines, which are potential vectors of ChD, especially in PESA neighborhoods.

In a study in a similar area, Jácome-Pinilla et al. [52] proved an actively dispersing area, and triatomines highly attracted to artificial lights. Furthermore, the environmental parameters encountered during this study, particularly during the first hours after sunset, are favorable for the active dispersal of sylvatic triatomines. One immediate recommendation is that external artificial lights on walls must remain turned off during the first hours after sunset, the period when most sylvatic. Triatomines find favorable atmospheric and environmental conditions for dispersal.

Entomological surveillance for triatomines in Barra do Garças has been done mainly by the population, showing the importance of passive surveillance by residents to detect foci of the triatomines. Passive surveillance improved risk management by the city health system, favoring timely intervention in the ChD transmission chain. This process is in agreement with entomological surveillance, which has been supported by community referees responsible for the Triatomine Information Post Network [38]. The population’s knowledge about triatomines and ChD is paramount to promoting collaboration in vector control and reducing vector transmission [53–55].

The primary limitation of this study was the COVID-19 pandemic. Because the domicile visits were reduced along with the communication of new triatomine specimens being caught, the number of specimens analyzed in 2020 was lower than in the other years. Another limiting factor was the number of dead triatomines delivered by residents, impairing the parasitological diagnosis of the presence of *T. cruzi* in these specimens.

## Conclusion

As far as we know, this study is the first to confirm the domiciliation of *T. williami* in urban areas of Barra do Garças, Mato Grosso, Brazil. This record occurred after sporadic invasions by this species in domiciles near an environmental conservation unit. This park is composed of native vegetation and wild animals, reservoirs for *T. cruzi*. These conditions reinforce the importance of entomological surveillance for the sanitary control of triatomines. Further studies are needed to better understand the ecology of these species for their prevention and control mechanisms, since they have a high natural infection rate by *T. cruzi*.

## Supplementary Information

**Additional file: Table S1.** Species of triatomines with a tendency toward the domiciliation process (presence of eggs/nymphs and adults) in human domiciles.

**Additional file 2: Figure S1.** Panoramic view of the Barra do Garças municipality and the PESA, Mato Grosso, Brazil.

**Additional file 3: Figure S2.** A. Adult female of *T. williami*; B. Head detail in the dorsal view of the specimens; C. wing venation pattern detail of the specimens; D. External female genitalia by dorsal view; E. General appearance of the connexivum chromatic pattern.

**Additional file 4: Figure S3.** The absolute and relative frequencies of triatomines collected in Barra do Garças, Mato Grosso, Brazil, from 2013 to 2020.

## Data Availability

Specimens from this study were deposited in the Herman Lent Collection of the Instituto Oswaldo Cruz in Rio de Janeiro, Brazil, and in the Entomological Collection Prof° Dr. José Maria Soares Barata, in the Faculty of Pharmaceutical Sciences UNESP—Araraquara—São Paulo, Brazil.

## Abbreviations

CTIOC: Collection of the Instituto Oswaldo Cruz
ChD: Chagas disease
CNPq: Conselho Nacional de Desenvolvimento Científico e Tecnológico
DTUs: Discrete typing units
ERSBG: Escritório Regional de Saúde de Barra do Garças
IBGE: Instituto Brasileiro de Geografia e Estatística
PCR: Polymerase chain reaction
PESA: Parque Estadual da Serra Azul
PCDCh: Chagas disease control program
SMS: Secretaria Municipal de Saúde
SESMT: Secretaria de Estado de Saúde de Mato Grosso
WHO: World Health Organization
