# Supplementary material for: Triatoma williami in intradomiciliary environments of urban areas in Mato Grosso State, Brazil: domiciliation process of a wild species?
Source: Infect Dis Poverty. 2022 Feb 14;11:18. doi: 10.1186/s40249-022-00938-4 (PMC8843021; doi:10.1186/s40249-022-00938-4)
Supplement: Supplementary file 1 — Additional file: Table S1. Species of triatomines with a tendency toward the domiciliation process (presence of eggs/nymphs and adults) in human domiciles. [file 40249_2022_938_MOESM1_ESM.docx]

Table 1. Species of triatomines with a tendency toward the domiciliation process (presence of eggs/nymphs and adults) in human domiciles.

| **Specie** | **City/State/Country** | **Reference** |
| --- | --- | --- |
| *Panstrongylus diasi* | Federal District, Brazil | Maeda *et al.* (2012) |
| *P. megistus* | São Paulo, Brazil | Silva e*t al.* (2021) |
|  | Federal District, Brazil | Maeda *et al.* (2012) |
| *P. rufotuberculatus* | La Gardenia, Colombia | Wolf and Castillo (2002) |
|  |  | Depickère et al. (2011) |
| *P. geniculatus* | Munecas, La Paz, Bolivia | Reyes-Lugo and Rodriguez-Acosta, (2000) |
| *Rhodnius neglectus* | Federal District, Brazil | Maeda *et al.* (2012) |
| *R. stali* | Alto Bebi, Bolivia | Matias *et al.* (2003) |
| *Triatoma brasiliensis macromelasoma* | São João do Piauí, Piauí, Brazil | Santos *et al*. (2017) |
| *T. rubrovaria* | Rio Grande do Sul, Brazil | Almeida *et al.* (2000)  CEVS/RS (2009) |
| *T. maculata* | Pitahaya,Venezuela | Reyes-Lugo and Reyes-Contreras (2011) |
|  | Boa Vista, RO, Brazil | Luitgards-Moura (2005) |
|  |  | Ricardo-Silva et al. (2016) |
|  |  |  |
| *T.nigromaculata* | Merida, Venezuela | Añez *et al.* (2005) |
| *T. pseudomaculata* | Minas Gerais, Brazil | Assis *et al.* (2007) |
| *T. sherlocki* | Bahia, Brazil | Almeida *et al.* (2009) |

Maeda MH, Knox MB, Gurgel -Gonçalves R. Occurrence of synanthropic triatomines (Hemiptera: Reduviidae) in the Federal District of Brazil. Rev Soc Bras Med Trop. 2012;45(1):71–6. [https://doi.org/10.1590/S0037-86822 012000100014](https://doi.org/10.1590/S0037-86822%20012000100014)

Silva RA, Virgino F, Estevão VAO, Martins ML, Duarte NA, et al. First report of colonization by Panstrongylus megistus (Burmeister, 1835) (Hemiptera, Reduviidae, Triatominae) in the Metropolitan Region of São Paulo, Brazil. Braz J Biol. 2021. <https://doi.org/10.1590/1519-6984.225562>

Wolff M, Castillo D. Domiciliation trend of *Panstrongylus rufotuberculatus* in Colombia. Mem Inst Oswaldo Cruz. 2002;97(3):297–300. https://doi. org/10.1590/S0074-02762002000300003

Depickére S, Durán P, Lopez R, Chávez T. Presença de colônias intradomi ‑ ciliares do inseto triatomíneo *Panstrongylus rufotuberculatus* em Muñecas, La Paz, Bolívia. Acta Trop. 2011;117(20):97–100. [https://doi.org/10.1016/j. actatropica.2010.10.005](https://doi.org/10.1016/j.%20actatropica.2010.10.005)

Reyes -Lugo M, Rodríguez -Acosta A. Domiciliation of selvatic Chagas disease vector *Panstrongylus geniculatus* Latreille 1811 (Triatominae: Reduviidae) in Venezuela. Trans R Soc Trop Med Hyg. 2000;94:508. https:// doi.org/10.1016/s0035-9203(00)90068 3 .

Matias A, de La Riva J, Martinez E, Torrez M, Dujardin JP. Domiciliation process of *Rhodnius stali* (Hemiptera: Reduviidae) in Alto Beni, La Paz, Bolivia. Trop Med Int Health. 2003;8(3):264–8. [https://doi.org/10.1046/j. 1365-3156.2003.01021.x](https://doi.org/10.1046/j.%201365-3156.2003.01021.x)

Santos MS, Sousa DM, Santos JP, Vieira JFPN, Gonçalves TCM, et al. Entomological survey in the state of Piauí, Northeastern Brazil, reveals intradomiciliary colonization of *Triatoma brasiliensis macromelasoma*. Rev Inst Med Trop Sao Paulo. 2017. [https://doi.org/10.1590/S1678-99462 01759027](https://doi.org/10.1590/S1678-99462%2001759027)

Almeida CE, Vinhaes MC, de Almeida JR, Silveira AC, Costa J. Monitoring the domiciliary and peridomiciliary invasion process of *Triatoma rubrovaria* in the State of Rio Grande do Sul, Brazil. Mem Inst Oswaldo Cruz 2000;95(6):761–8. <https://doi.org/10.1590/S0074-02762000000600003>

CEVS/Rio Grande do Sul. Bol Epidemiol. 2009; 11(3).

Reyes -Lugo M, Reyes -Contreras M, Salvi I, Gelves W, Avilán A, Llavaneras D, et al. The association of Triatoma maculata (Ericsson 1848) with the gecko *Thecadactylus rapicauda* (Houttuyn 1782) (Reptilia: Squamata: Gek ‑ konidae): a strategy of domiciliation of the Chagas disease peridomestic vector in Venezuela? Asian Pac J Trop Biomed. 2011;1(4):279–84. https:// doi.org/10.1016/S2221-1691(11)60043 9

Luitgards -Moura JF, Vargas AB, Almeida CA, Magno -Esperança G, Agapito -Souza R, Folly -Ramos E, et al. A *Triatoma maculata* (Hemiptera, Reduviidae, Triatominae) populations from Roraima, Amazon region, Bra ‑ zil, hassome bionomic characteristic of a potential Chagas disease vector. Rev Inst Med Trop. 2005;47:131–7. [https://doi.org/10.1590/S0036-46652 005000300003](https://doi.org/10.1590/S0036-46652%20005000300003)

Ricardo -Silva A, Gonçalves TCM, Luitgards -Moura JF, Lopes CM, da Silva SP, Bastos AQ, et al. *Triatoma maculata* colonises urban domicilies in Boa Vista, Roraima, Brazil. Mem Inst Oswaldo Cruz. 2016;111(11):703–6. <https://doi.org/10.1590/0074-02760160026>

Añez N, Crisante G, Agustina R, Diaz N, Diaz S, Lizano E, et al. Domicili ‑ ación de *Triatoma nigromaculata* de la región montana del sur de Mérida, Venezuela. Bol Malariol Salud Ambient. 2005;45(1):47–8.

Assis GLM, Azeredo BVM, Carbajal de la Fuente AL, Diotaiuti L, de Lana M. Domiciliation of *Triatoma pseudomaculata* (Corrêa e Espínola 1964) in the Jequitinhonha Valley, State of Minas Gerais. Rev Soc Bras Med Trop. 2007;40(4):391–6. <https://doi.org/10.1590/S0037-86822007000400003>

Almeida CE, Folly -Ramos E, Peterson AT, Lima -Neiva V, Gumiel M, Duarte R, et al. Could the bug *Triatoma sherlock* I be vectoring Chagas disease in small mining communities in Bahia, Brazil? Med Vet Entomol. 2009;23:410–7. <https://doi.org/10.1111/j.1365-2915.2009.00822.x>
