# Supplementary figures and images for: Triatoma williami in intradomiciliary environments of urban areas in Mato Grosso State, Brazil: domiciliation process of a wild species?
Source: Infect Dis Poverty. 2022 Feb 14;11:18. doi: 10.1186/s40249-022-00938-4 (PMC8843021; doi:10.1186/s40249-022-00938-4)

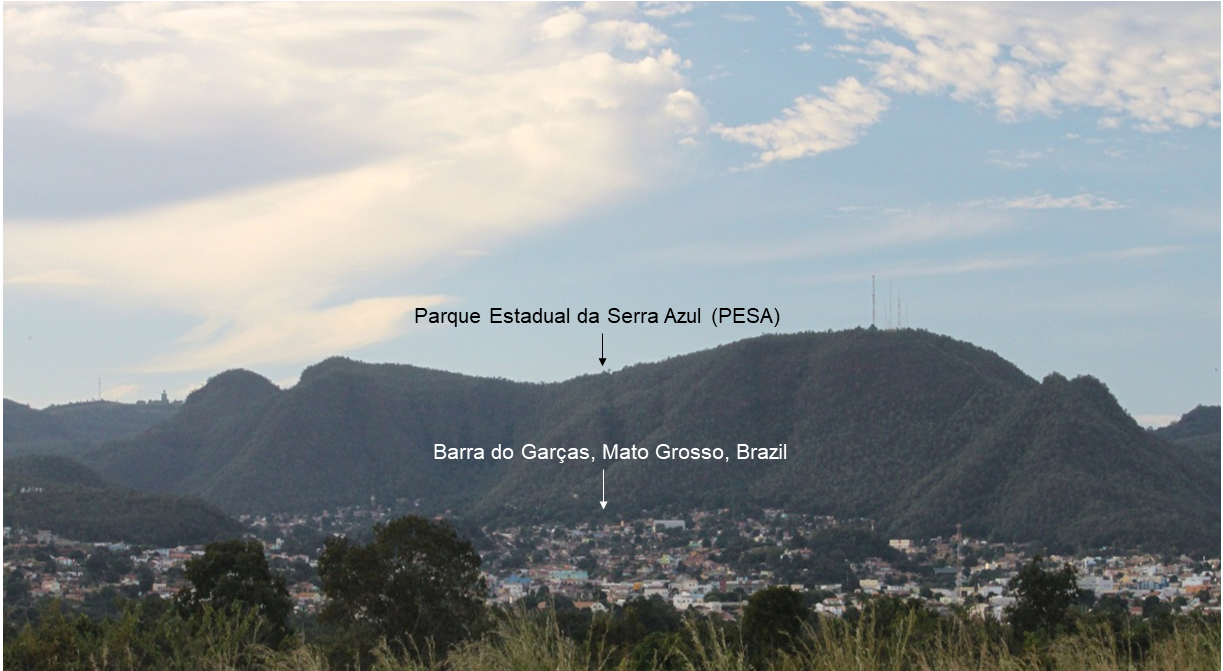

Supplement: Supplementary file 2 — Additional file 2: Figure S1. Panoramic view of the Barra do Garças municipality and the PESA, Mato Grosso, Brazil. [file 40249_2022_938_MOESM2_ESM.tif]

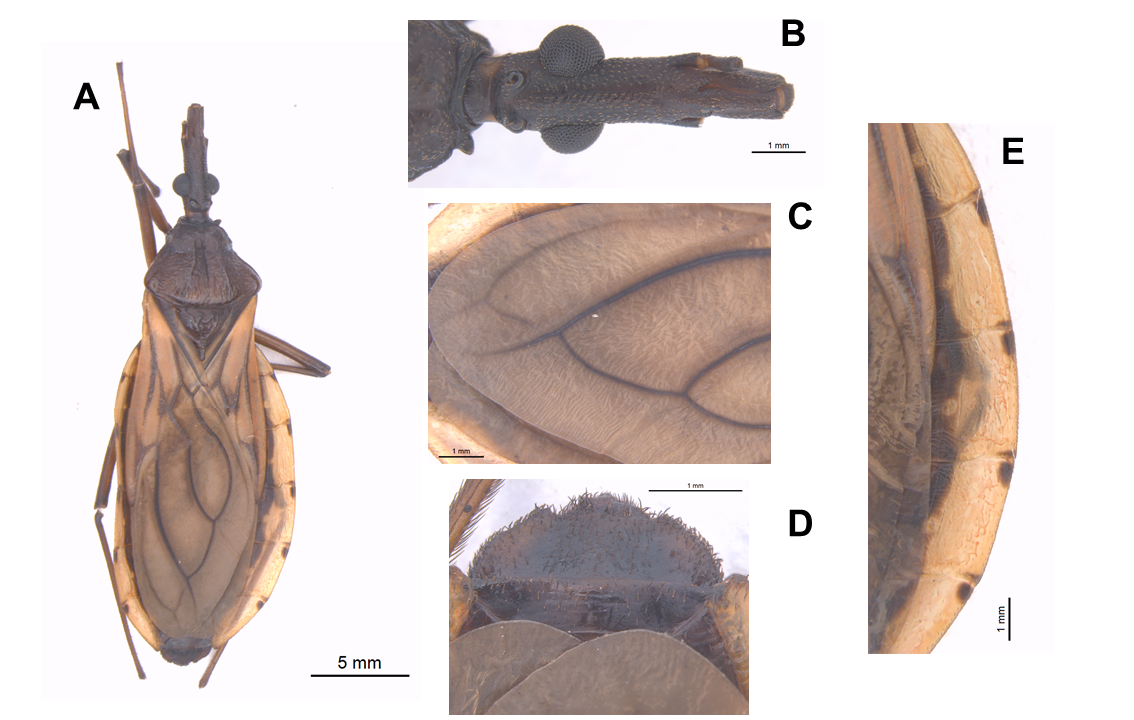

Supplement: Supplementary file 3 — Additional file 3: Figure S2. A. Adult female of T. williami; B. Head detail in the dorsal view of the specimens; C. wing venation pattern detail of the specimens; D. External female genitalia by dorsal view; E. General appearance of the connexivum chromatic pattern. [file 40249_2022_938_MOESM3_ESM.tif]

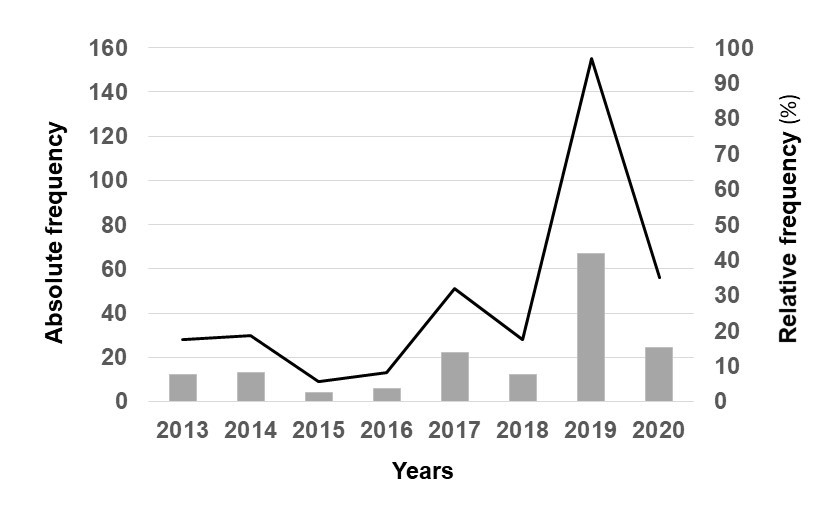

Supplement: Supplementary file 4 — Additional file 4: Figure S3. The absolute and relative frequencies of triatomines collected in Barra do Garças, Mato Grosso, Brazil, from 2013 to 2020. [file 40249_2022_938_MOESM4_ESM.jpg]
